# The admission level of CRP during cardiogenic shock is a strong independent risk marker of mortality

**DOI:** 10.1038/s41598-024-67556-y

**Published:** 2024-07-16

**Authors:** François Roubille, Miloud Cherbi, Eran Kalmanovich, Quentin Delbaere, Eric Bonnefoy-Cudraz, Etienne Puymirat, Guillaume Schurtz, Edouard Gerbaud, Laurent Bonello, Pascal Lim, Guillaume Leurent, Camille Roubille, Clément Delmas

**Affiliations:** 1 PhyMedExp, Université de Montpellier, INSERM, CNRS, Cardiology Department, CHU de Montpellier, Montpellier, France; 2 Intensive Cardiac Care Unit, Rangueil University Hospital, 31059 Toulouse, France; 3 Institute of Metabolic and Cardiovascular Diseases (I2MC), UMR-1048, National Institute of Health and Medical Research (INSERM), Toulouse, France; 4 Cardiac Intensive Care Unit, Division of Cardiology, Shamir Medical Center, Affiliated to Tel Aviv University Faculty of Medicine, Tel Aviv, Israel; 5 Intensive Cardiac Care Unit, Lyon Brom University Hospital, Lyon, France; 6 Department of Cardiology, Assistance Publique-Hôpitaux de Paris (AP-HP), Hôpital Européen Georges Pompidou, 75015 Paris, France; 7 Université de Paris, 75006 Paris, France; 8 Urgences Et Soins Intensifs de Cardiologie, CHU Lille, University of Lille, Inserm U1167, 59000 Lille, France; 9 Intensive Cardiac Care Unit and Interventional Cardiology, Hôpital Cardiologique du Haut Lévêque, 5 Avenue de Magellan, 33604 Pessac, France; 10 Bordeaux Cardio-Thoracic Research Centre, U1045, Bordeaux University, Hôpital Xavier Arnozan, Avenue du Haut Lévêque, 33600 Pessac, France; 11 Aix-Marseille Université, 13385 Marseille, France; 12 Intensive Care Unit, Department of Cardiology, Assistance Publique-Hôpitaux de Marseille, Hôpital Nord, 13385 Marseille, France; 13 Mediterranean Association for Research and Studies in Cardiology (MARS Cardio), Marseille, France; 14 Intensive Cardiac Care Unit, Cardiology Department, Henri Mondor University Hospital, AP-HP, Créteil, France; 15 Department of Cardiology, CHU Rennes, Inserm, LTSI—UMR 1099, Univ Rennes 1, 35000 Rennes, France; 16 Internal Medicine Department, Montpellier University Hospital, Montpellier, France; 17 Intensive Care Unit, Cardiology Department, University Hospital of Montpellier, 34295 Montpellier, France

**Keywords:** Cardiogenic shock, Inflammation, CRP, Infection, Epidemiology, Prognosis, Mortality, Cardiology, Prognostic markers, Biomarkers, Outcomes research

## Abstract

Inflammatory processes are involved not only in coronary artery disease but also in heart failure (HF). Cardiogenic shock (CS) and septic shock are classically distinct although intricate relationships are frequent in daily practice. The impact of admission inflammation in patients with CS is largely unknown. FRENSHOCK is a prospective registry including 772 CS patients from 49 centers. One-month and one-year mortalities were analyzed according to the level of C-reactive protein (CRP) at admission, adjusted on independent predictive factors. Within 406 patients included, 72.7% were male, and the mean age was 67.4 y ± 14.7. Four groups were defined, depending on the quartiles of CRP at admission. Q1 with a CRP < 8 mg/L, Q2: CRP was 8–28 mg/L, Q3: CRP was > 28–69 mg/L, and Q4: CRP was > 69 mg/L. The four groups did not differ regarding main baseline characteristics. However, group Q4 received more often antibiotics in 47.5%, norepinephrine in 66.3%, and needed more frequently respiratory support and renal replacement therapy. Whether at 1 month (P_trend_ = 0.01) or 1 year (P_trend_ < 0.01), a strong significant trend towards increased all-cause mortality was observed across CRP quartiles. Specifically, compared to the Q1 group, Q4 patients demonstrated a 2.2-fold higher mortality rate at 1-month (95% CI 1.23–3.97, *p* < 0.01), which persisted at 1-year, with a 2.14-fold increase in events (95% CI 1.43–3.22, *p* < 0.01). Admission CRP level is a strong independent predictor of mortality at 1 month and 1-year in CS. Specific approaches need to be developed to identify accurately patients in whom inflammatory processes are excessive and harmful, paving the way for innovative approaches in patients admitted for CS.

NCT02703038.

## Introduction

Inflammatory processes play a pivotal role in the pathophysiological aspects of cardiovascular diseases encompassing^1^, acute conditions such as acute coronary syndromes (ACS)^2^ and chronic states typified as stable coronary artery disease (CAD)^3^. These insights have given rise to novel prospects for therapeutic interventions within the cardiovascular domain^4^. Additionally, inflammation exerts a significant influence on the progression of heart failure (HF), instigating processes that encompass fibrosis, apoptosis, and tissue or cellular dysfunctions^5,6^. This pivotal role of inflammation underscores its relevance in both acute and chronic cardiovascular conditions, accentuating the imperative for targeted therapeutic strategies.

Cardiogenic shock (CS) is a complex heterogeneous clinical syndrome characterized by hypotension and hypoperfusion primarily attributed to reduced cardiac output resulting mainly from myocardial dysfunction^7^. Despite advancements in medical care, CS remains associated with a notably high mortality rate, approaching 50–60% at the end of one year, as documented in prior registries^8,9^. While ACS is a well-recognized cause of CS, CS can stem from various etiologies in clinical practice. Septic shock is a severe and life-threatening condition characterized by a dysregulated host response to infection, resulting in widespread systemic inflammation, organ dysfunction, and profound hypotension^10^. By contrast septic shock is triggered by a severe infection, leading to acute and severe inflammation and responsible for multiorgan failure (MOF)^11^. CS and septic shock share then intricate pathophysiological pathways, as can both ultimately lead to MOF. Despite their conventional differentiation, their interrelatedness in clinical practice is intricate and not easily distinguishable. The hypothesis posits that inflammation may exert a significant role in the pathogenesis of CS. However, the precise mechanisms by which inflammation contributes to CS in patients predominantly afflicted with CS, as opposed to septic shock, remains unclear. This knowledge gap argues for further exploration to comprehensively comprehend the underpinning of CS, particularly in cases where septic etiologies are less pronounced.

While acute inflammation in the initial stages of CS is widely acknowledged as a predictor of poorer prognosis, worsening both short- and long-term mortality^12^, limited understanding exists regarding the influence of basal inflammation levels at admission. Moreover, the existing data primarily pertain to CS occurring as a complication of ACS^7^.

This study aims to investigate the association between baseline inflammation levels upon admission and the mortality of individuals admitted for CS, using a comprehensive analysis from in a nationwide CS registry.

## Materials and methods

### Patient population

As previously reported^9^, the FRENSHOCK study constitutes a prospective, observational, and multicenter survey, conducted from April to October 2016. It included 772 patients admitted for CS across various intensive care/intensive cardiac care units (ICU/ICCU) across various healthcare institutions in France, spanning from primary to tertiary centers, university, and non-university, and public and private healthcare facilities.

All adult patients (≥ 18 years old) with CS were prospectively included in this registry if they met at least one criterion of each of the following three components: (1) Low cardiac output defined by low SBP < 90 mmHg and/or the need for maintenance with vasopressors/inotropes and/or a low cardiac index < 2.2 L/min/m^2^; (2) Left and/or right heart filling pressure elevation, defined by clinical signs, radiology, blood tests (brain natriuretic peptide [BNP] > 400 pg/mL and/or N-terminal-pro hormone BNP [NT-proBNP] > 900 pg/mL)", echocardiography, or signs of invasive hemodynamic overload and (3) Signs of organ malperfusion, which could be clinical (oliguria, confusion, pale and/or cold extremities, mottled skin) and/or biological (lactate > 2 mmol/L, metabolic acidosis, renal failure, liver insufficiency). The presence of at least one criterion from each of these three categories was mandatory for the diagnosis.

For each patient, investigators had to specify one to three triggers among the following: ischemic (type 1 or 2 acute myocardial infarction [AMI]), mechanical complications (valvular injury, ventricular septal defect), ventricular and supraventricular arrhythmia, severe bradycardia, iatrogenesis (medication induced), infections, non-observance of previous medication.

Additionally, investigators were encouraged to provide optional biological parameters including serum CRP levels, often accessible in routine clinical practice.

### Data collection

The data collection protocol has been previously published elsewhere^9^. In brief, the data acquisition process encompassed the gathering of comprehensive medical history, previous treatments, in-hospital CS management [inotropes/vasopressors, mechanical ventilation, and acute mechanical circulatory support (aMCS)], clinical, biological, and echocardiographic parameters (at admission and at 24h).

As the SCAI SHOCK Stage Classification^13^ was not yet available at the FRENCSHOCK registry, we retrospectively determined the maximum SCAI classification stage reached during hospitalization based on the total use of vasopressors, inotropes, and aMCS devices as previously described by Thayer et al.^14^. Briefly, Stage A represents patients at risk for CS, which was not applicable to our study population. Stage B encompasses patients with early symptoms who do not require pharmacological or mechanical support. Stage C includes patients with hypoperfusion requiring initial intervention with either one drug or one MCS device. Stage D refers to patients whose condition worsens despite initial intervention, necessitating additional drugs or MCS treatment. Lastly, Stage E identifies patients who deteriorate further and require maximal support, defined as needing at least 2 MCS devices and 2 drugs during hospitalization.

### Follow-up

All-cause mortality was assessed at one month and one year. The primary endpoint was 1-year all-cause mortality. Secondary endpoint was 1-month all-cause mortality.

### Ethics

The study was conducted in accordance with the Helsinki Declaration and French law. Written informed consent was obtained for all patients. Recorded data and their storage were approved by the CCTIRS (French Health Research Data Processing Advisory Committee) (n° 15.897) and the CNIL (French Data Protection Agency) (n° DR-2016-109).

### Statistical analysis

Continuous variables were reported as means and standard deviation (SD) or medians and interquartile ranges (IQR) when appropriate. Categorical variables were described as frequencies and percentages. The overall population was divided into quartiles based on the level of CRP on admission. Quantitative variables were compared by the Kruskal–Wallis test; post hoc comparison was done using the Dunn’s test with Bonferroni correction to determine differences between groups. Categorical variables were compared by the Pearson chi-square test or, when indicated, the Fisher exact test, and adjusted Bonferroni post hoc testing was performed in case of significant overall difference. To analyse trends across quartiles, we employed the Cochran–Armitage test for qualitative variables and the Jonckheere–Terpstra test for quantitative variables, both described in the text and tables as “P_trend_”. To identify independent predictors for each outcome, we employed a multivariate stepwise logistic regression approach. Initially, univariate logistic regression analyses assessed the association of all baseline characteristics (age, sex ratio, body mass index, cardiovascular risk factors, comorbidities, New York Heart Association [NYHA] functional class, history of previous heart disease, initial cardiac arrest, sinus rhythm), CS' triggers (ischemic, mechanical complication, ventricular and supraventricular arrhythmia, infections, non-observance, iatrogenesis), and markers of CS severity (left ventricle ejection fraction [LVEF] ≤ 30%, lactates ≥ 4 mmol/L, estimated glomerular filtration rate (eGFR) ≤ 30 mL/min) with each primary and secondary outcome. Subsequently, based on their statistical significance in univariate analyses, and their clinical relevance, a backward reduction process was applied to include only characteristics with *p* ≤ 0.05 in the multivariable models for adjusted outcome analyses. Variance inflation factor (VIF) analysis was used to assess collinearity between variables, with a threshold set at 5. Primary outcome of all-cause mortality was assessed using Kaplan–Meier time-to-event analysis, and Cox proportional hazards models were used to determine the adjusted hazard ratio (aHR), 95% confidence interval (CI) and *p* values. We have strengthened the interpretation of the results by calculating the restricted mean survival times (RMST). Main analysis was a comparison between CS patients depending on the quartiles of CRP at admission. Sensitivity analysis was conducted by excluding patients with sepsis trigger to account for confounding biases and assess the robustness of the results. In addition, a Cox model with restricted cubic spline (RCS) functions was employed to determine the optimal predictive cut-off point for CRP and to assess the shape of the associations between CRP levels (as a continuous measure) and all-cause mortality. Potential nonlinearity was evaluated using a likelihood ratio test, comparing the model with only a linear term to the model including both linear and cubic spline terms.

All tests were two-tailed. A value of *p* ≤ 0.05 was accepted as statistically significant. Analyses were performed using R software [version 4.1.2 (2021-11-01)].

## Results

### Baseline patients’ characteristics

**Figure 1:**
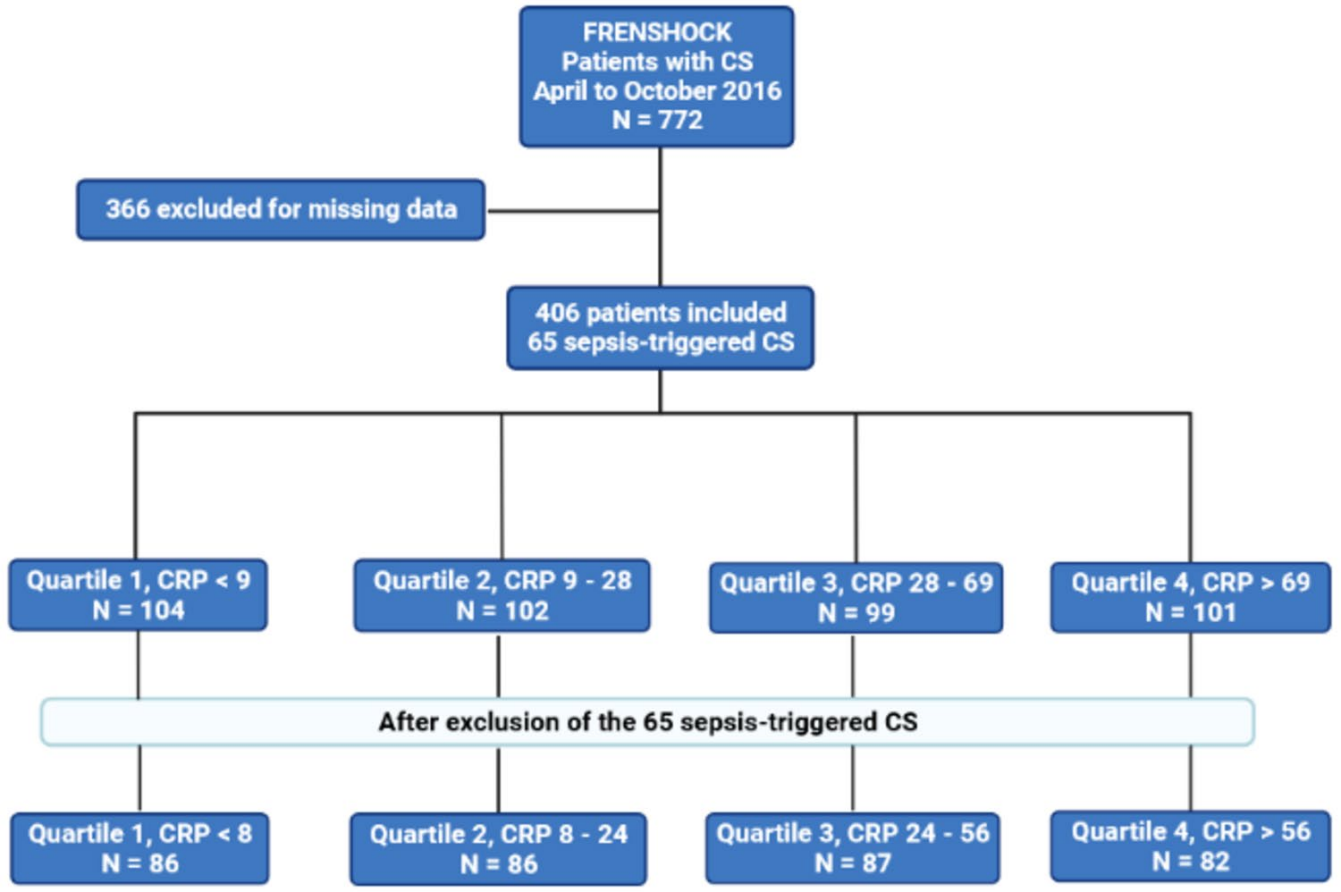
Flow chart of the study.

As shown in Fig. 1, our analysis encompassed 406 out of 772 CS patients from a total of 49 centers. 366 patients were excluded from the analysis due to missing data related to CRP levels at admission. Our analysis categorized patients into four distinct groups based on the CRP levels quartiles at admission: Quartile 1 (Q1) group with a CRP < 8 mg/L, Quartile 2 (Q2) group with CRP levels ranging from 8 to 28 mg/L, Quartile (Q3) group with CRP levels ranging from > 28 to 69 mg/L, and Quartile (Q4) with CRP > 69 mg/L.

Table 1 provides a comprehensive overview of the baseline characteristics of studies cohort. The mean age was 67.4 (± 14.7) years, with predominance of male individuals (72.7%).

**Table 1:** Baseline characteristics according to initial CRP.*

	Overall population (n = 406)	Quartile 1 (< 9) (n = 104)	Quartile 2 (9–28) (n = 102)	Quartile 3 (28–69) (n = 99)	Quartile 4 (> 69) (n = 101)	*p* value	P_trend_
Age, mean ± SD, years	67.4 ± 14.7	67.8 ± 13.8	67.2 ± 14.3	67.1 ± 17.3	67.5 ± 13.3	0.97	0.83
Male, n (%)	295 (72.7)	80 (76.9)	74 (72.5)	72 (72.7)	69 (68.3)	0.59	0.19
Body mass index, mean ± SD, kg/m^2^	25.6 ± 5.3 (n = 393)	25.7 ± 4.3 (n = 100)	25.2 ± 5 (n = 99)	25.9 ± 6.6 (n = 95)	25.4 ± 5.2 (n = 99)	0.53	0.38
Risk factors, n (%)							
Diabetes mellitus	121 (29.8)	29 (27.9)	32 (31.4)	30 (30.3)	30 (29.7)	0.96	0.82
Hypertension	200 (49.3)	55 (52.9)	48 (47.1)	50 (50.5)	47 (46.5)	0.78	0.48
Dyslipidemia	142 (35.0)	34 (32.7)	37 (36.3)	35 (35.4)	36 (35.6)	0.95	0.70
Current smoker	100 (25.4) (n = 393)	33 (32.4) (n = 102)	26 (26.0) (n = 100)	22 (22.9) (n = 96)	19 (20.0) (n = 95)	0.22	0.04
Medical history, n (%)							
Peripheral artery disease	51 (12.6)	8 (7.7)	14 (13.7)	13 (13.1)	16 (15.8)	0.34	0.10
Chronic kidney disease	91 (22.4)	17 (16.3)	33 (32.4)	25 (25.3)	16 (15.8)	0.01	0.66
COPD	26 (6.4)	4 (3.8)	7 (6.9)	10 (10.1)	5 (5.0)	0.29	0.54
ICD	75 (18.5)	20 (19.2)	26 (25.5)	15 (15.2)	14 (13.9)	0.14	0.13
Active cancer	29 (7.1)	3 (2.9)	6 (5.9)	8 (8.0)	12 (11.9)	0.08	0.01
Stroke	29 (7.1)	5 (4.8)	9 (8.8)	10 (10.1)	5 (5.0)	0.34	0.87
Previous PCI	94 (23.2)	27 (26)	28 (27.5)	22 (22.2)	17 (16.8)	0.28	0.08
NYHA functional status, n (%)							
≥ 3	170 (42.8) (n = 397)	36 (35) (n = 103)	60 (60.6) (n = 99)	41 (42.3) (n = 97)	33 (33.7) (n = 98)	< 0.01	0.35
History of cardiac disease, n (%)							
All causes	249 (61.3)	58 (55.8)	76 (74.5)	62 (62.6)	53 (52.5)	< 0.01	0.33
Ischemic	134 (33)	32 (30.8)	39 (38.2)	34 (34.3)	29 (28.7)	0.49	0.64
Hypertrophic	6 (1.5)	3 (2.9)	1 (1.0)	2 (2.0)	0 (0.0)	0.35	0.15
Toxic	18 (4.4)	2 (1.9)	11 (10.8)	1 (1.0)	4 (4.0)	< 0.01	0.71
Dilated	41 (10.1)	7 (6.7)	14 (13.7)	15 (15.2)	5 (5.0)	0.04	0.78
Valvular	37 (9.1)	8 (7.7)	12 (11.8)	8 (8.1)	9 (8.9)	0.74	0.99
Hypertensive	14 (3.4)	3 (2.9)	3 (2.9)	4 (4.0)	4 (4.0)	0.95	0.59
Previous medications, n (%)							
Aspirin	161 (39.6)	45 (43.3)	39 (38.2)	37 (37.4)	40 (39.6)	0.83	0.58
P2Y12 inhibitors	82 (20.2)	25 (24.0)	19 (18.6)	20 (20.2)	18 (17.8)	0.69	0.33
Vitamin K antagonist	90 (22.2)	22 (21.2)	26 (25.5)	25 (25.3)	17 (16.8)	0.40	0.48
Direct oral anticoagulant	36 (8.9)	8 (7.7)	11 (10.8)	11 (11.1)	6 (5.9)	0.51	0.70
ACE inhibitors	162 (39.9)	42 (40.4)	39 (38.2)	41 (41.4)	40 (39.6)	0.97	0.97
Sacubitril/valsartan	10 (2.6) (n = 390)	1 (1) (n = 101)	6 (6.3) (n = 96)	2 (2.1) (n = 95)	1 (1.0) (n = 98)	0.06	0.59
Statins	159 (39.2)	43 (41.3)	46 (45.1)	36 (36.4)	34 (33.7)	0.34	0.14
Betablockers	179 (44.1)	45 (43.3)	47 (46.1)	47 (47.5)	40 (39.6)	0.69	0.67
Loop diuretics	221 (54.4)	47 (45.2)	62 (60.8)	60 (60.6)	52 (51.5)	0.07	0.39
Aldosterone antagonist	67 (16.5)	12 (11.5)	23 (22.5)	21 (21.2)	11 (10.9)	0.04	0.86
Thiazide diuretics	23 (5.8) (n = 400)	6 (5.8)	8 (7.9) (n = 101)	4 (4.2) (n = 96)	5 (5.1) (n = 99)	0.70	0.58
Non-dihydropyridine CCB	7 (1.7) (n = 401)	1 (1.0)	3 (3.0) (n = 101)	1 (1.0) (n = 96)	2 (2.0) (n = 100)	0.67	0.83
Amiodarone	76 (19.1) (n = 397)	13 (12.5)	25 (25.3) (n = 99)	15 (16.1) (n = 93)	23 (22.8) (n = 101)	0.08	0.20
Other anti-arrhythmic	19 (4.8) (n = 398)	5 (4.9) (n = 103)	5 (5.0) (n = 101)	6 (6.3) (n = 96)	3 (3.1) (n = 98)	0.78	0.67
SCAI stage, n (%)						0.06	
B	38 (9.4)	10 (9.7)	11 (10.8)	11 (11.1)	6 (5.9)		0.41
C	146 (36.0)	45 (43.3)	37 (36.3)	31 (31.3)	33 (32.7)		0.08
D	213 (52.5)	43 (41.3)	53 (52.0)	57 (57.6)	60 (59.4)		< 0.01
E	9 (2.2)	6 (5.8)	1 (1.0)	0 (0.0)	2 (2.0)		0.06

*ACE* angiotensin-converting enzyme, *BB* betablockers, *CCB* calcium channel blocker, *COPD* chronic obstructive pulmonary disease, *ICD* implantable cardioverter-defibrillator, *NYHA* New York Heart Association, *PCI* percutaneous coronary intervention, *SD* standard deviation.

Notably, the four quartiles groups exhibited no significant differences in primary characteristics, including age, cardiovascular risk factors (e.g., diabetes, chronic obstructive pulmonary disease which were present in 29.8% and 6.4% of the entire analyzed population, respectively), except for a trend towards an increasing prevalence of active cancers from Q1 to Q4 (P_trend_ = 0.01). Based on our adapted classification, 38 patients (9.4%) were categorized as SCAI shock stage B, 146 (36.0%) as SCAI stage C, 213 (52.5%) as SCAI stage D, and 9 (2.2%) as SCAI stage E, with a significant trend towards an increase in the proportion of stage D across the quartiles (P_trend_ < 0.01). Differences in initial clinical presentation were observed between the groups, particularly with regard to NYHA classification in Q2 group, where 60.6% of patients present NYHA III or IV, approximately double the proportion observed in Q1 and Q4 groups (*p* < 0.05 for each). CAD served as the primary cause of CS in one-third of cases and was evenly distributed among quartiles, certain rare etiologies displayed variations among the groups. Regarding previous medications, there were no significant differences among the groups regarding statins (39.2% in the overall population), which are known for their pleiotropic anti-inflammatory effects and other medications indicated in chronic HF, including beta-blockers (44.1% in the overall population), angiotensin-converting enzyme inhibitors (39.9% of the entire population). Sacubitril–valsartan was rarely administered. The only medication that observed to have a non-uniform administration was aldosterone antagonists albeit with relatively low overall usage (16.5% of the whole population). The administration of anticoagulants or antiplatelet agents was equally distributed among the groups.

### CS and infections could be intricated

Table 2 illustrates the various triggers identified in the study cohort. Ischemic etiology was predominant, accounting for more than one-third of cases, followed by ventricular and/or supraventricular arrhythmias, which were reported in approximately one-quarter to one-third of patients.

**Table 2:** Distribution of cardiogenic shock triggers between groups.

	Quartile 1 (< 9) (n = 104)	Quartile 2 (9–28) (n = 102)	Quartile 3 (28–69) (n = 99)	Quartile 4 (> 69) (n = 101)	*p* value	P_trend_
Ischemic, n (%)	46 (44.2)	27 (26.5)	27 (27.3)	40 (39.6)	0.01	0.52
Supraventricular tachycardia, n (%)	11 (10.6)	18 (17.6)	18 (18.2)	9 (8.9)	0.12	0.78
Infectious disease, n (%)	7 (6.7)	10 (9.8)	14 (14.1)	34 (33.7)	< 0.01	< 0.01
Ventricular arrhythmia, n (%)	20 (19.2)	8 (7.8)	11 (11.1)	8 (7.9)	0.03	0.03
Iatrogenesis, n (%)	3 (2.9)	12 (11.8)	5 (5.1)	5 (5.0)	0.047	0.98
Non-observance, n (%)	2 (1.9)	3 (2.9)	2 (2.0)	3 (3.0)	0.94	0.74
Mechanical complications, n (%)	3 (2.9)	2 (2.0)	2 (2.0)	7 (6.9)	0.17	0.13
Conduction disorder, n (%)	4 (3.8)	2 (2.0)	1 (1.0)	1 (1.0)	0.41	0.12

Notably, the incidence of severe infections was noteworthy and demonstrated a progressive increase across CRP quartiles (P_trend_ < 0.01) ranging from 6.7% in Q1 group to 33.7% in Q4 groups, as anticipated. Conversely, ventricular arrhythmias exhibited a progressively decreasing prevalence, from 19.2% in Q1 to 7.9% in Q4 (P_trend_ < 0.01).

### CS presentation and prognostic markers in the four quartiles of CRP

As delineated in Table 3, the four quartiles stratified by CRP levels upon admission exhibited discernible variations with respect to certain prognostic indicators. It is important to note that dissimilarities did not consistently manifest in relation to the fourth quartile (Q4).

**Table 3:** Clinical, echocardiographic, and laboratory parameters according to initial CRP.

	Quartile 1 (< 9) (n = 104)	Quartile 2 (9—28) (n = 102)	Quartile 3 (28—69) (n = 99)	Quartile 4 (> 69) (n = 101)	p value	P_trend_
Clinical presentation at admission						
Heart rate, mean ± SD, bpm	94.5 ± 31.8	88.5 ± 24.8	98.1 ± 29.3	103.3 ± 31.2 (n = 100)	< 0.01	< 0.01
SBP, mean ± SD, mmHg	105.3 ± 30.8	95.7 ± 21.8	105.0 ± 24.5	98.1 ± 23.9 (n = 100)	0.01	0.51
DBP, mean ± SD, mmHg	64.8 ± 18.8	59.3 ± 14.4	65.8 ± 19.2	60.1 ± 15.4 (n = 100)	0.07	0.47
MBP, mean ± SD, mmHg	77.0 ± 20.4	70.9 ± 15.0 (n = 101)	78.5 ± 19.2	71.7 ± 17.2 (n = 100)	0.02	0.52
Sinus rhythm, n (%)	49 (47.1)	63 (61.8)	45 (45.5)	49 (49.0) (n = 100)	0.08	0.65
Skin mottling, n (%)	30 (34.5) (n = 87)	32 (34.4) (n = 93)	28 (30.8) (n = 91)	37 (45.7) (n = 81)	0.21	0.22
Left heart failure, n (%)	71 (68.3)	79 (77.5)	78 (78.8)	75 (74.3)	0.31	0.31
Right heart failure, n (%)	49 (47.1)	68 (66.7)	61 (61.6)	51 (50.5)	0.01	0.80
Cardiac arrest, n (%)	13 (12.5)	4 (3.9)	4 (4.0)	4 (4.0)	0.02	0.02
Blood tests at admission, median						
(IQR)						
Sodium, mmol/L	137.0 (134.0–140.0)	134.0 (131.0–137.8)	136.0 (131.5–140.0)	134.0 (130.0–137.0)	< 0.01	< 0.01
Potassium, mmol/L	4.0 (3.7–4.9) (n = 94)	4.2 (4.0–5.0) (n = 95)	4.4 (4.0–5.0) (n = 90)	4.0 (4.0–5.0) (n = 94)	0.02	0.28
Creatinin, μmol/L	121.0 (93.0–153.8)	142.0 (108.3–246.3)	146.0 (104.0–205.5)	140.0 (101.0–214.0)	< 0.01	0.03
Bilirubin, mg/L	12.0 (7.8–20.4) (n = 86)	22.0 (12.0–37.5) (n = 81)	20.0 (12.5–35.5) (n = 83)	16.5 (9.9–29.0) (n = 84)	< 0.01	0.04
Haemoglobin, g/dL	13.3 (12.0–15.0) (n = 102)	12.5 (11.0–14.0)	13.0 (11.0–14.0)	11.9 (10.0–13.3)	< 0.01	< 0.01
Arterial blood lactates, mmol/L	3.0 (2.0–5.8) (n = 92)	2.6 (1.7–4.0) (n = 95)	3.0 (2.0–5.0) (n = 88)	3.0 (2.0–4.9) (n = 93)	0.045	0.68
ASAT, UI/L	65.0 (37.0–175.0) (n = 85)	45.0 (29.0–110.5) (n = 91)	117.5 (48.0–700.0) (n = 80)	121.0 (55.0–428.5) (n = 83)	< 0.01	< 0.01
ALAT, UI/L	51.0 (26.0–112.8) (n = 90)	34.5 (20.8–121.8) (n = 92)	60.0 (28.0–578.5) (n = 83)	57.0 (30.5–183.3) (n = 84)	0.01	0.04
PT, %	67.0 (46.0–88.0) (n = 101)	52.0 (35.0–75.0) (n = 100)	49.0 (28.5–68.5) (n = 95)	59.0 (43.0–70.0) (n = 93)	< 0.01	0.01
Nt-proBNP, pg/mL	5,445.0 (2,000.0–11,797.0) (n = 41)	12,420.5 (6,456.0–22,702.5) (n = 36)	10,743.5 (4,887.0–23,887.0) (n = 42)	18,897.0 (6,564.0–35,000).0 (n = 41)	< 0.01	< 0.01
BNP, pg/mL	991.0 (314.5–1,456.5) (n = 44)	1,973.5 (876.3–3,471.5) (n = 40)	1,545.5 (575.0–4,248.5) (n = 42)	1,334.0 (762.0–2,834.0) (n = 37)	< 0.01	0.09
Baseline echocardiography						
LVEF, mean ± SD, %	29.1 ± 14.5	25.6 ± 13.1	24.3 ± 12.8 (n = 98)	29.1 ± 13.5 (n = 97)	< 0.01	0.81
TAPSE, median (IQR), mm	14.0 (10.8–18.3) (n = 40)	11.0 (9.0–16.0) (n = 45)	14.0 (11.0–16.0) (n = 34)	11.0 (9.0–14.5) (n = 38)	0.12	0.7
PSVtdi, median (IQR), cm/s	8.0 (6.0–10.8) (n = 30)	8.0 (6.0–11.0) (n = 33)	7.5 (7.0–9.0) (n = 32)	9.0 (6.0–12.0) (n = 31)	0.49	0.54
Severe mitral regurgitation, n (%)	14 (13.9) (n = 101)	27 (27.8) (n = 97)	16 (17) (n = 94)	8 (8.4) (n = 95)	< 0.01	0.12
Severe aortic stenosis, n (%)	5 (4.8)	5 (4.9)	5 (5.2) (n = 97)	3 (3.1) (n = 98)	0.89	0.59
Severe aortic regurgitation, n (%)	2 (2.0) (n = 102)	2 (2.0) (n = 101)	1 (1.0) (n = 96)	2 (2.0) (n = 97)	0.94	0.91

*ALAT* alanine aminotransferase, *ASAT* aspartate aminotransferase, *BB* betablockers, *BNP* Brain natriuretic peptide, *CRP* C-reactive protein, *DBP* diastolic blood pressure, *IQR* interquartile range, *LVEF* left ventricular ejection fraction, *MBP* mean blood pressure, *Nt-proBNP* N-terminal-pro hormone BNP, *PSVtdi* peak systolic velocity tissue Doppler imaging, *PT* prothrombin time, *SBP* systolic blood pressure, *SD* standard deviation, *TAPSE* tricuspid annular plane systolic excursion.

According to pairwise analyses Q4 patients demonstrated a notably elevated cardiac rate in comparison to Q2 (103.3 ± 31.2 beats per minute [bpm] versus 88.5 ± 24.8 bpm, *p* < 0.01), with a trend of increasing rates across quartiles (P_trend_ < 0.01). Furthermore, the Q4 category exhibited a significantly diminished serum sodium level when compared with either Q1 or Q3 patients. Similarly, Q4 group displayed a significantly reduced hemoglobin level when juxtaposed with all other quartile groups, confirmed by a significant decreasing trend across quartiles (P_trend_ < 0.01). Conversely, no statistically significant distinctions were observed among quartile groups concerning lactate levels.

Additionally, it was noteworthy that Q4 patients manifested a markedly elevated concentration of NT-proBNP in comparison to the Q1 category (18,897; 6564–35,000 ng/L versus 5445; 2000–11,797 ng/L), corroborated by a significant increasing trend across quartiles (P_trend_ < 0.01). Intriguingly, despite this elevated NT-proBNP level, Q4 patients concurrently exhibited a significantly greater LVEF when contrasted with the Q3 category.

### In-hospital management according to the four quartiles of CRP

The in-hospital management revealed notable disparities, particularly for Q4 patients (Table 4). Significant distinctions were observed in medication usage, notably with respect to norepinephrine administration. Notably, patients falling within the Q4 category were administered norepinephrine at a significantly higher frequency compared to those in Q1 (66.3% vs. 45.2%, respectively, *p* = 0.02, P_trend_ < 0.01). Additionally, patients in the Q4 category, by necessity, received respiratory support more frequently than those in Q2 (43.6% vs. 22.5%, *p* = 0.01), and approximately twice as often necessitated renal replacement therapy, which was subject to increasingly frequent use across quartiles (P_trend_ < 0.01). Lastly, patients in Q4 were more frequently subjected to antibiotic treatment (47.5%), a statistically significant increase compared to Q1 (26.5%, *p* < 0.01) and Q2 (15.8%, *p* < 0.01).

**Table 4:** In-hospital management according to initial CRP.

	Overall population (n = 406)	Quartile 1 (< 9) (n = 104)	Quartile 2 (9–28) (n = 102)	Quartile 3 (28–69) (n = 99)	Quartile 4 (> 69) (n = 101)	*p* value	P_trend_
Medications used, n (%)							
Dobutamine Norepinephrine Epinephrine Levosimendan Loop diuretics Thiazide diuretics Aldosterone antagonist	326 (80.3) 218 (53.7) 49 (12.1) 37 (9.1) 270 (72.2) (n = 374) 18 (4.9) (n = 367) 56 (15.0) (n = 374)	83 (79.8) 47 (45.2) 18 (17.3) 9 (8.7) 65 (65.0) (n = 100) 3 (3.1) (n = 98) 14 (14.0) (n = 100)	80 (78.4) 53 (52.0) 8 (7.8) 13 (12.7) 72 (75.8) (n = 95) 6 (6.4) (n = 94) 18 (18.9) (n = 95)	83 (83.8) 51 (51.5) 10 (10.1) 11 (11.1) 75 (80.6) (n = 93) 6 (6.7) (n = 90) 16 (17.2) (n = 93)	80 (79.2) 67 (66.3) 13 (12.9) 4 (4.0) 58 (67.4) (n = 86) 3 (3.5) (n = 85) 8 (9.3) (n = 86)	0.78 0.02 0.18 0.15 0.058 0.55 0.29	0.84 < 0.01 0.43 0.22 0.48 0.82 0.38
Number of vasopressors/inotropes used, n (%)						0.21	
0	40 (9.9)	10 (9.6)	12 (11.8)	12 (12.1)	6 (5.9)		0.42
1	151 (37.2)	46 (44.2)	36 (35.3)	31 (31.3)	38 (37.6)		0.26
2	169 (41.6)	33 (31.7)	46 (44.2)	44 (44.4)	46 (45.5)		0.06
3	43 (10.6)	15 (14.4)	6 (5.9)	12 (12.1)	10 (9.9)		0.58
4	3 (0.7)	0 (0.0)	2 (2.0)	0 (0.0)	1 (0.9)		0.78
Respiratory support, n (%)							
Non-invasive	86 (21.2)	22 (21.2)	12 (11.8)	27 (27.3)	25 (24.8)	0.04	0.15
Invasive	133 (32.8)	38 (36.5)	23 (22.5)	28 (28.3)	44 (43.6)	< 0.01	0.20
Acute MCS, n (%)							
Overall	58 (14.3)	19 (18.3)	12 (11.8)	12 (12.1)	15 (14.9)	0.52	0.52
IABP	19 (4.7)	7 (6.7)	2 (2.0)	3 (3.0)	7 (6.9)	0.22	0.87
Impella ECLS Renal replacement therapy, n (%) Volume expander, n (%) Isotonic saline Balanced solutions Glucose solution Macromolecules Any PCI, n (%)	11 (2.7) 34 (8.4) 61 (15.0) 146 (36.0) 97 (23.9) 18 (4.4) 60 (14.8) 14 (3.4) 117 (66.9) (n = 175)	4 (3.8) 11 (10.6) 12 (11.5) 38 (36.5) 23 (22.1) 7 (6.7) 17 (16.3) 3 (2.9) 41 (74.5) (n = 55)	3 (2.9) 8 (7.8) 13 (12.7) 30 (29.4) 20 (19.2) 2 (2.0) 14 (13.7) 3 (2.9) 22 (56.4) (n = 39)	2 (2.0) 7 (7.1) 8 (8.1) 34 (34.3) 21 (21.2) 5 (5.1) 16 (16.2) 3 (3.0) 22 (59.5) (n = 37)	2 (2.0) 8 (7.9) 28 (27.7) 44 (43.6) 33 (32.7) 4 (4.0) 13 (12.9) 5 (5.0) 32 (72.7) (n = 44)	0.82 0.82 < 0.01 0.21 0.17	0.36 0.47 < 0.01 0.23 0.08 0.56 0.61 0.44 0.81
Anti-infectious therapies, n (%)							
Antibiotics	125 (33.0) (n = 379)	26 (26.5) (n = 98)	15 (15.8) (n = 95)	39 (42.9) (n = 91)	45 (47.4) (n = 95)	< 0.01	< 0.01
Antifungal agents	9 (2.5) (n = 367)	1 (1.0) (n = 99)	1 (1.1) (n = 93)	2 (2.2) (n = 89)	5 (5.8) (n = 86)	0.13	0.03

*ECLS* extracorporeal life support, *IABP* intra-aortic balloon pump, *MCS* mechanical circulatory support, *PCI* percutaneous coronary intervention.

### CS evolution according to quartiles of CRP

**Figure 2:**
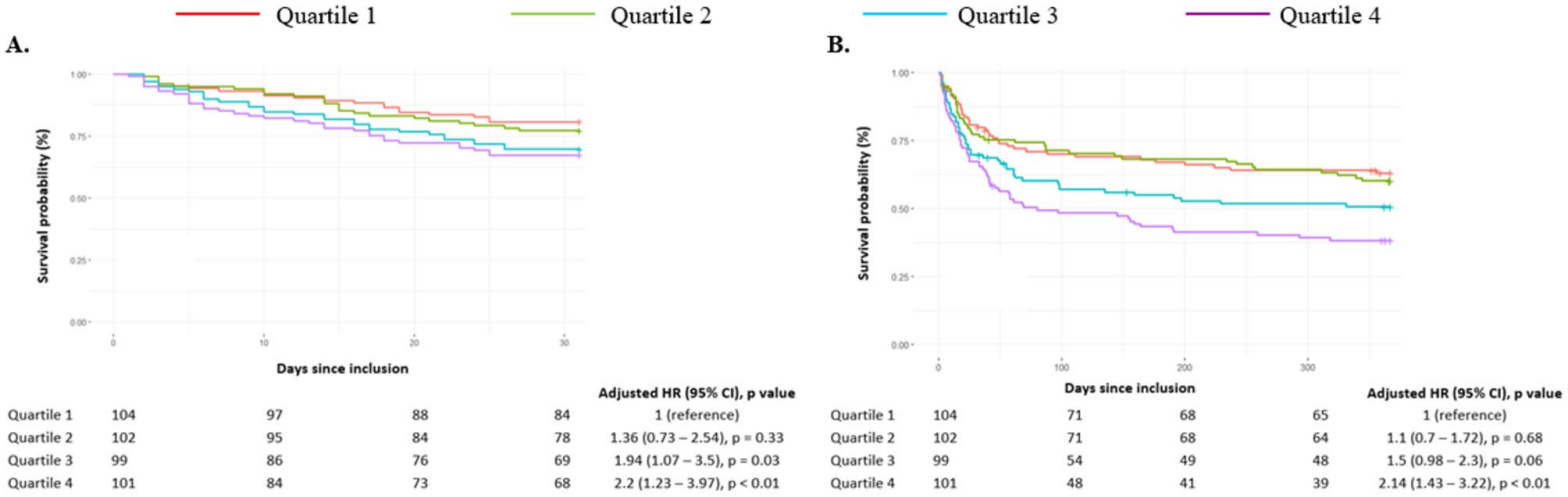
Short- and long-term mortality outcomes after CS according to baseline CRP. Panel A represents 1-month overall mortality. Panel B focus on 1-year mortality. The cumulative incidences of 1-year and 1-month mortality were estimated with the use of the Kaplan–Meier method; hazard ratios and 95% confidence intervals were estimated with the use of Cox regression models. According to significant characteristics found as independent predictive factors in multivariable analyses, 1-year mortality was adjusted for age, previous PCI, SVT trigger, iatrogenic trigger. 1-month mortality was adjusted for age and current smoking. CS, cardiogenic shock; PCI, percutaneous coronary intervention; SVT, supra-ventricular tachycardia.

In the context of multivariate analysis, considering significant independent predictive factors, adjustments were made for 1-month mortality with respect to age and current smoking. Conversely, for 1-year mortality adjustments were made for age, prior percutaneous coronary intervention (PCI), triggers related to sustained ventricular tachycardia, and iatrogenic triggers. As shown in Fig. 2*,* one-month outcomes are presented in panel A, reflecting one-month overall mortality, and in panel B showing one-year mortality. First, whether at 1 month (P_trend_ = 0.01) or 1 year (P_trend_ < 0.01), a strong significant trend towards increased all-cause mortality was observed across CRP quartiles, suggesting a linear correlation between increased quartiles of CRP level and all-cause mortality. Of note, 1-month RMST gradually decreased from 27.5 days for the Q1 group to 24.5 days for the Q4 group (respectively 27.1 and 25.4 days for Q2 and Q3). For 1-year mortality, they ranged from 254 days in the Q1 group to 174 days in the Q4 group (respectively 255 and 211 days for Q2 and Q3).

Specifically, compared to the Q1 group (taken as reference), Q4 patients demonstrated a 2.2-fold higher mortality rate at one month (95% CI 1.23–3.97, *p* < 0.01), which persisted at one year, with a 2.14-fold increase in events (95% CI 1.43–3.22, *p* < 0.01). The Q3 group also exhibited increased mortality at 1 month (aHR 1.94 [95% CI 1.07–3.5], *p* = 0.03), with a suggestive trend at 1 year but not reaching significance (aHR 1.50 [95% CI 0.98–2.30], *p* = 0.06). Interestingly, Q1 and Q2 groups, representing patients with CRP levels below 28 mg/L appear to have similar event rates both in the short term and at one-year. Unadjusted hazard ratios are presented for comparison in *Supplementary Table 1*, also showing an increased mortality at both 1-month and 1-year in Q4. Of note, no collinearity was observed for any covariates, with VIF values ranging from 1.04 to 1.63.

### Restricted cubic spline curves

**Figure 3:**
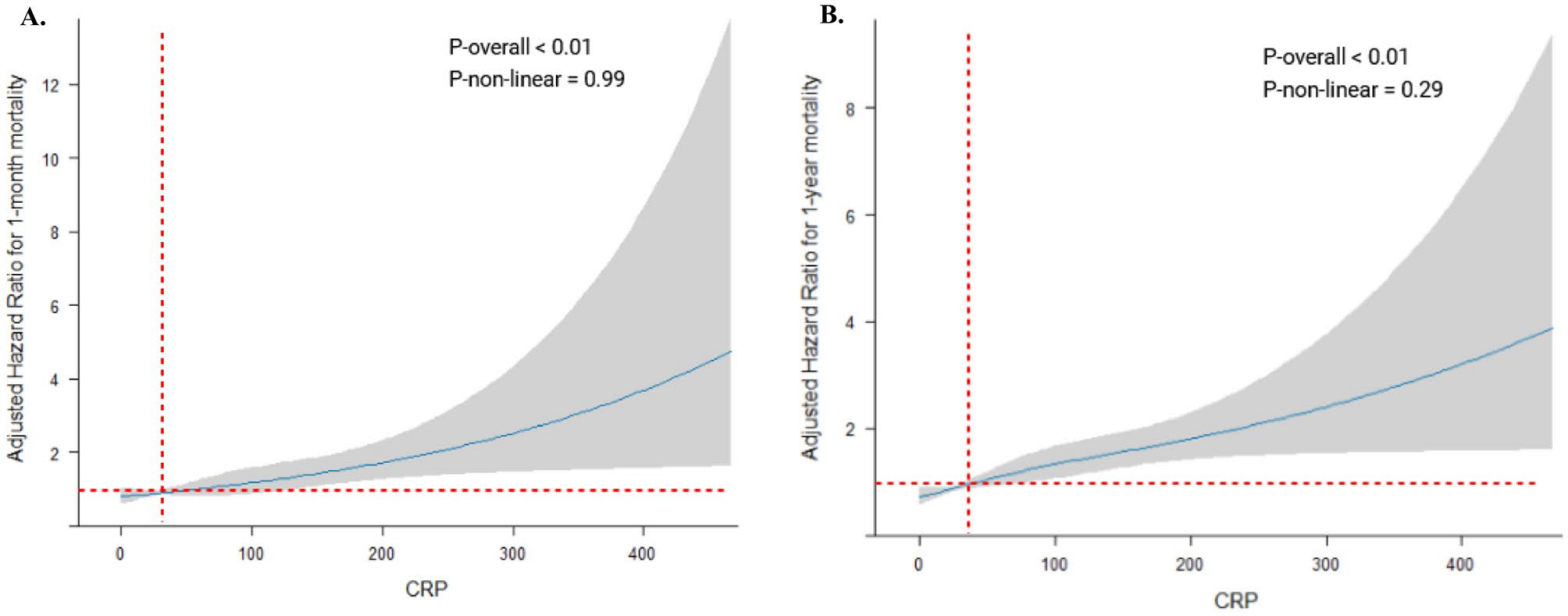
Restricted cubic spline curves for the relationship between CRP and all-cause mortality. (**A**) Represents the restricted cubic spline curve for 1-month mortality, adjusted for age and current smoking. (**B**) Represents the restricted cubic spline curve for 1-year mortality, adjusted for age, previous PCI, SVT trigger, iatrogenic trigger.

Monotonic relationships between CRP level and all-cause mortality were confirmed by spline analysis results, with P-overall < 0.01 for both 1-month and 1-year mortality (Fig. 3). After adjustment for potential confounding factors, the optimal cut-off for CRP's impact on 1-month mortality was 32.0 mg/L and 41.0 mg/L for 1-year mortality. Besides, the p-value for non-linearity was higher than 0.05, suggesting a possible linear association between CRP and mortality.

### Sensitivity analysis after exclusion of patients with *sepsis* trigger

Similarly, no differences were observed in most baseline characteristics, except for a higher proportion of chronic kidney disease in the Q2 group (31.4%, nearly double compared to Q1 and Q4, *p* < 0.01 for each), which also consistently had the highest proportion of NYHA stage ≥ 3 (56.6%). A total of 36 patients (10.6%) were categorized as SCAI shock stage B, 134 (39.3%) as SCAI stage C, 162 (47.5%) as SCAI stage D, and 9 (2.6%) as SCAI stage E, with no significant difference in the distribution of SCAI stages between the groups. Sensitivity analysis also produced consistent results with the main analyses across quartiles regarding a trend towards increased creatinine levels (P_trend_ = 0.03), bilirubin (P_trend_ < 0.01), and NtproBNP (P_trend_ < 0.01), along with a decreasing trend for haemoglobin (P_trend_ < 0.01) and tricuspid annular plane systolic excursion (TAPSE) (P_trend_ = 0.03). Finally, a significant increase in mortality at 1 month was observed for the Q3 (aHR 2.14 [95% CI 1.09–4.20], *p* = 0.03) and Q4 (aHR 1.97 [95% CI 1.01–3.85], *p* = 0.047) groups, associated with significant excess mortality at 1 year for Q4 (aHR 1.71 [95% CI 1.08–2.72], *p* = 0.02), overall supported by a statistically significant trend for mortality at 1 year (P_trend_ < 0.01).

All data reflecting the analyses conducted after exclusion of patients with sepsis triggers are presented in *Supplementary Tables 2, 3, 4 and Supplementary Fig. 1*.

## Discussion

In this large national registry analysis, we demonstrate that baseline inflammation, as indicated by CRP levels upon **admission in ICU/ICCU, offers valuable prognostic insights for patients admitted with CS.** Patients presenting with highest levels of CRP, particularly in the Q4 with CRP exceeding 68 mg/L, exhibited a 2.2 folds increase in 1-month mortality, (95% CI 1.23–3.97, *p* < 0.01) and a 2.14 folds increase in 1-year mortality (95% CI 1.43–3.22, < 0.01) compared to patients with lower levels of CRP on admission (as briefly summarized in supplementary Fig. 2). Conversely, patients with CRP levels below than median (approximately 30 mg/L, as commonly observed in clinical practice) display significantly favorable outcomes. These findings remained robust even after a sensitivity analysis excluding patients with septic trigger, suggesting that inflammatory processes may contribute to the complex pathophysiological pathways underpinning CS. This aligns with the conventional concept of CS as a vicious circle wherein hemodynamic deterioration triggers MOF, which in turn, may exacerbate inflammation.

Given the involvement of inflammation in pathophysiology, addressing harmful mechanisms like bacterial translocations, iatrogenic infections or thromboses warrants consideration. However, it remains challenging to determine whether exclusively targeting inflammatory processes would be effective or safe, given that inflammation serves important physiological functions. This prompts serval key questions. First, can inflammatory biomarkers assist in making informed clinical decisions? Ongoing trials (like (NCT05748860) aim to evaluate the utility of various biomarkers, including those related to inflammation.

Secondly, whether it is feasible to target inflammatory processes as a therapeutic target remains largely unknown. Recent research suggested that inflammation is not merely a marker of disease severity but may also play an active role. This concept has been explored in fields like atherosclerosis or stable conditions for decades, paving the way for investigations into the therapeutic potential of anti-inflammatory or immunomodulatory drugs^15^.

In line with this, several clinical trials are presently underway. For example, tocilizumab, an anti-interleukin-6 therapy, is evaluated in 100 patients admitted with AMI complicated by CS (NCT05350592). This study utilizes a four-arm design with a surrogate endpoint (NT-proBNP). Another trial (NCT05642273), is testing a specific membrane capable of absorbing cytokine and lipopolysaccharide in 60 patients requiring veno-arterial extracorporeal membrane oxygenation. Its goal is to reduce inflammatory biomarkers. A large trial conducted in France which enrolled 380 CS, has recently been completed^16^. In this study, patients were randomized into the treatment group (hydrocortisone (50 mg intravenous bolus every 6 h) and fludrocortisone (50 μg once a day enterally) for 7 days or until discharge) and control group, with a focus on targeting pathophysiological pathways, including inflammation. This trial is expected to provide valuable insights, particularly regarding mechanisms common to CS and infections that have been targeted for intervention.

Infections and CS may share complex interactions, and their individual roles in certain patients remain unclear. Initial clinical evaluation is challenging, exemplified by Q1 group with low CRP levels, sometimes associated with infections. Similar observation apply to Q2 group (CRP < 28 mg/L). In contrast, the Q4 group, as expected exhibited higher infections prevalence (33.7%), but also a significant proportion of ischemic causes (39.6%). This study, revealed widespread antibiotics use, even in patients without evident inflammation or infection. The frequent use of invasive ventilation (e.g., 36.5% in the Q1 group) may explain the extensive antibiotic utilization.

Beyond infections, mounting evidence suggests that **inflammation plays a role in acute conditions**, especially in AMI, possibly acting as a marker and even a contributor to **myocardial dysfunction**^17,18^. Prior research has long implicated CRP as a factor of myocardial injury^19^. Trials, such as the CANTOS trial^20^, have been designed based on minimal inflammation assessed by high-sensitivity CRP, demonstrating the potential efficacy of anti-inflammatory agents like canakinumab in patients with stable CAD. However, implementing such therapeutic innovations in routine clinical practice raised concerns. In contrast, the COLCOT trial^2^, administered the pleiotropic anti-inflammatory agent colchicine to post-AMI patients regardless of their initial inflammatory status, offering a systematic approach for candidate treatment in patients without active infection or other contraindications. The question of whether CRP or alternative patient selection criteria can enhance the effectiveness of these approaches warrants further investigation.

This challenge is compounded by the dynamic nature of **the interrelationships,** with parameters that may rapidly change within days or even hours, influenced by confounding factors and treatments. Notably, 24 h after than the initial assessment, all groups exhibited increased of CRP levels, but only the first three groups demonstrated statistically significant elevations (data not shown). This implies a common trend toward heightened inflammation among these groups, with Q4 group possibly indicating a more severe condition due to earlier involvement of inflammatory processes.

From a clinical perspective with practical implications, **we might categorize patients into two distinct populations**: the first group consists of patients with CRP levels below the critical threshold of 32 mg/L (for 1-month mortality) and 41 mg/L (for 1-year mortality) determined via RCS regression, where we did not observe any excess mortality. In contrast, the second population, comprising patients with CRP levels above these cut-offs, including a large proportion of patients from the Q3 group (CRP > 28 mg/L) and all patients from the Q4 group (CRP > 70 mg/L), demonstrates worse outcomes.

In clinical setting, this stratification could prove valuable for early patient risk assessment. It supports the rationale for conducting trials targeting anti-inflammatory interventions in these patients’ populations, especially the second group. Additionally, it underscores the potential utility of incorporating CRP levels stratification into trial design, facilitating the investigation of pleiotropic, rapidly acting anti-inflammatory drugs such as colchicine, corticoids, or mildly immunosuppressive drugs, as well as specific absorption or other innovating approaches.

Beyond the initial outcomes, patients in Q4 group exhibited a higher incidence of adverse events, even after one-year follow-up. This suggests that proinflammatory processes may actively contribute to detrimental pathophysiological mechanisms, **not only at the acute phase but also at later stages**. These findings support the consideration of similar approaches during the follow-up assessments, such as, at one or three months post-admission to the intensive care unit. Firstly, given that approximately half of the patients have ischemic cardiopathy, it is advisable to implement established and recommended anti-inflammatory medications, notably colchicine as recommended^21^. Secondly, the implementation of dedicated patient visits and proactive management programs should be considered as a part of overarching strategy to address potential proinflammatory cofactors effectively.

Our findings align with recent research that has established the significance of baseline inflammation in patients experiencing AMI complicated by CS, as reported in study conducted at two center over an extended time frame^12^. In this study, we contribute consistent data from a nationwide registry, which offers several strengths. First, our registry encompasses a diverse range of healthcare setting, including public university hospitals, non-university hospitals and private healthcare facilities, providing a comprehensive representation of CS cases. Secondly, our recruitment period was relatively short, minimizing the potential for variations in clinical practice and influenced by seasonal fluctuations on infectious cases. Importantly, our study encompasses all types of CS, not limited to AMI, broadening its clinical relevance.

Furthermore, recent randomized control study had demonstrated the limited utility of extracorporeal life support (ECLS) in patients with CS^22,23^. The authors proposed that inflammatory processes may contribute to this unfavourable outcome. However, it's important to acknowledge that our findings only suggest a potential association, as other factors such as bleeding complications, infection, and disease progression are also well-documented in these patients and undoubtedly contribute to the increased mortality they experience. Further studies are warranted to ascertain both the efficacy and potential risks associated with specific interventions in patients with active inflammatory pathophysiological pathways.

### Limitations

First, it is important to note that while the collection of CRP data was feasible, it was not obligatory. Consequently, the presence of missing data resulted in the exclusion of 47% of the initially enrolled patients. Nevertheless, this exclusion did not impede our ability to delineate distinct quartiles characterized by specific features and disparate clinical outcomes, thereby strengthening the validity of our analytical approaches. Furthermore, it would be interesting to broaden the scope of biological parameters (such as procalcitonin) and/or clinical markers intended to establish and more closely stratify the degree of inflammation. Regrettably, this was not feasible due to insufficient data availability.

Secondly, we acknowledge the potential existence of mixed shocks as infections was reported in 65 patients included our analyses, constituting approximately 16% of the analyzed population. However, it is noteworthy that the reports of infections by the investigators lacked specific related descriptions, suggesting a higher likelihood of co-infections or iatrogenic infections, such as those arising from mechanical ventilation or peripheral access. It is important to clarify that patients with septic shock were typically not included in our analysis unless CS was the primary presentation. Furthermore, we attempted to mitigate the impact of this bias by conducting a sensitivity analysis excluding patients with septic triggers. This analysis confirmed the presence of a strong and gradual relationship between CRP levels at admission and short- and long-term mortality.

## Conclusion

The presence of inflammation upon admission for CS emerges as a strong independent prognostic indicator for mortality, evidence at both at 1-month and 1-year follow up intervals. Consequently, there is a compelling need to formulate precise methodologies for the accurate identification of individuals in whom excessive and detrimental inflammatory processes are at play. These imperative highlights the promising avenues for innovative interventions aimed at mitigating inflammation at the time of admission, and extending to after discharge, for patients admitted with CS.

### Supplementary Information

Supplementary Information 1.

Supplementary Information 2.

## Data Availability

The datasets used and/or analysed during the current study available from the corresponding author on reasonable request.

## Abbreviations

ACS: Acute coronary syndrome
aHR: Adjusted hazard ratio
ALAT: Alanine aminotransferase
AMI: Acute myocardial infarction
aMCS: Acute mechanical circulatory support
ASAT: Aspartate aminotransferase
BNP: Brain natriuretic peptide
CCTIRS: Comité consultatif pour le traitement de l'information en matière de recherche dans le domaine de la santé
CAD: Coronary artery disease
CCB: Calcium channel blocker
CI: Confidence interval
CNIL: Commission nationale de l'informatique et des libertés
COPD: Chronic obstructive pulmonary disease
CRP: C-reactive protein
CS: Cardiogenic shock
DBP: Diastolic blood pressure
ECLS: Extracorporeal life support
eGFR: Estimated glomerular filtration rate
HF: Heart failure
HR: Hazard ratio
IABP: Intra-aortic balloon pump
ICD: Implantable cardioverter-defibrillator
ICU: Intensive care unit
ICCU: Intensive cardiac care unit
IQR: Interquartile range
LVEF: Left ventricle ejection fraction
MBP: Mean blood pressure
MOF: Multiorgan failure
NYHA: New York Heart Association
Nt-proBNP: N-terminal-pro hormone BNP
PCI: Percutaneous coronary intervention
PSVtdi: Peak systolic velocity tissue Doppler imaging
PT: Prothrombin time
Q: Quartile
RMST: Restricted mean survival times
RRT: Renal replacement therapy
SBP: Systolic blood pressure
SD: Standard deviation
SVT: Supraventricular tachycardia
TAPSE: Tricuspid annular plane systolic excursion
