# Supplementary material for: The admission level of CRP during cardiogenic shock is a strong independent risk marker of mortality
Source: Sci Rep. 2024 Jul 16;14:16338. doi: 10.1038/s41598-024-67556-y (PMC11252392; doi:10.1038/s41598-024-67556-y)
Supplement: Supplementary file 1 — Supplementary Information 1. [file 41598_2024_67556_MOESM1_ESM.docx]

**Supplementary Table 1.** Adjusted and unadjusted hazard ratios for 1-month and 1-year mortality in the overall population.

**Supplementary Table 2.** Baseline characteristics according to initial CRP after exclusion of sepsis-triggered CS.

**Supplementary Table 3.** Clinical, echocardiographic, and laboratory parameters according to initial CRP after exclusion of sepsis-triggered CS.

**Supplementary Table 4.** In-hospital management according to initial CRP after exclusion of sepsis-triggered CS.

**Supplementary Figure 1.** Short- and long-term mortality outcomes after CS according to baseline CRP after exclusion of sepsis-triggered CS.

|  | **1-month mortality** | | **1-year mortality** | |
| --- | --- | --- | --- | --- |
|  | **Unadjusted HR (95% CI), p value** | **Adjusted HR (95% CI), p value** | **Unadjusted HR (95% CI), p value** | **Adjusted HR (95% CI), p value** |
| Quartile 1 | 1 (reference) | 1 (reference) | 1 (reference) | 1 (reference) |
| Quartile 2 | 1.20 (0.66 – 2.18), p = 0.56 | 1.36 (0.73 – 2.54), p = 0.33 | 1.08 (0.69 – 1.69), p = 0.73 | 1.1 (0.7 – 1.72), p = 0.68 |
| Quartile 3 | 1.69 (0.96 – 2.97), p = 0.07 | 1.94 (1.07 – 3.5), p = 0.03 | 1.48 (0.97 – 2.28), p = 0.07 | 1.5 (0.98 – 2.3), p = 0.06 |
| Quartile 4 | 1.89 (1.08 – 3.29), p = 0.03 | 2.2 (1.23 – 3.97), p < 0.01 | 2.06 (1.38 – 3.09), p < 0.01 | 2.14 (1.43 – 3.22), p < 0.01 |

**Supplementary Table 1. Adjusted and unadjusted hazard ratios for 1-month and 1-year mortality in the overall population.**

HR = hazard ratio

| **Supplementary Table 2. Baseline characteristics according to initial CRP after exclusion of sepsis-triggered CS.**  ***** | | | | | | | |  |
| --- | --- | --- | --- | --- | --- | --- | --- | --- |
|  | **Overall population  (n = 341)** | **Quartile 1  (< 8) (n = 86)** | **Quartile 2  (8 - 24) (n = 86)** | **Quartile 3  (24 - 56) (n = 87)** | **Quartile 4  (> 56) (n = 82)** | **p value** | **P_trend_** |  |
| Age, mean ± SD, years  Male, n (%)  Body mass index, mean ± SD, kg/m²  Risk factors, n (%)  Diabetes mellitus  Hypertension  Dyslipidemia  Current smoker  Medical history, n (%)  Peripheral artery disease  Chronic kidney disease  COPD  ICD  Active cancer  Stroke  Previous PCI  NYHA functional status, n (%)  ≥ 3  History of cardiac disease, n (%)  All causes  Ischemic  Hypertrophic  Toxic  Dilated  Valvular  Hypertensive  Previous medications, n (%)  Aspirin  P2Y12 inhibitors  Vitamin K antagonist  Direct oral anticoagulant  ACE inhibitors  Sacubitril/valsartan  Statins  Betablockers  Loop diuretics  Aldosterone antagonist  Thiazide diuretics  Non-dihydropyridine CCB  Amiodarone  Other anti-arrhythmic  SCAI stage, n (%)  B  C  D  E  CS triggers, n (%)  Ischemic  Supraventricular tachycardia  Ventricular arrhythmia  Iatrogenesis  Non-observance  Mechanical complications  Conduction disorder | 67.8 ± 14.5  247 (72.4)  25.6 ± 5.2 (n = 328)  102 (29.9)  175 (51.3)  116 (34.0)  84 (25.3) (n = 332)  42 (12.3)  74 (21.7)  19 (5.6)  63 (18.5)  18 (5.3)  25 (7.3)  75 (22.0)  147 (44.3) (n = 332)  204 (59.8)  111 (32.6)  5 (1.5)  16 (4.7)  35 (10.3)  31 (9.1)  12 (3.5)  137 (40.2)  69 (20.2)  70 (20.5)  33 (9.7)  133 (39)  10 (3.1) (n = 326)  132 (38.7)  148 (43.4)  187 (54.8)  55 (16.1)  15 (4.5) (n = 335)  7 (2.1) (n = 336)  67 (20.2) (n = 332)  18 (5.3) (n = 337)  36 (10.6)  134 (39.3)  162 (47.5)  9 (2.6)  130 (38.1)  52 (15.2)  46 (13.5)  20 (5.9)  9 (2.6)  12 (3.5)  7 (2.1) | 67.0 ± 13.8  67 (77.9)  25.8 ± 4.1 (n = 82)  23 (26.7)  44 (51.2)  28 (32.6)  32 (38.1) (n = 84)  8 (9.3)  11 (12.8)  3 (3.5)  15 (17.4)  2 (2.3)  4 (4.7)  21 (24.4)  29 (34.1) (n = 85)  45 (52.3)  26 (30.2)  2 (2.3)  2 (2.3)  6 (7.0)  7 (8.1)  3 (3.5)  37 (43)  22 (25.6)  14 (16.3)  7 (8.1)  34 (39.5)  1 (1.2) (n = 83)  34 (39.5)  34 (39.5)  36 (41.9)  8 (9.3)  4 (4.7)  1 (1.2)  11 (12.8)  4 (4.7)  10 (11.6)  34 (39.5)  36 (41.9)  6 (7.0)  40 (46.5)  9 (10.5)  18 (20.9)  3 (3.5)  2 (2.3)  3 (3.5)  4 (4.7) | 67.2 ± 14.3  64 (74.4)  25.2 ± 5 (n = 84)  29 (33.7)  44 (51.2)  30 (34.9)  19 (22.4) (n = 85)  12 (14.0)  27 (31.4)  7 (8.1)  23 (26.7)  5 (5.8)  9 (10.5)  21 (24.4)    47 (56.6) (n = 83)  59 (68.6)  32 (37.2)  1 (1.2)  8 (9.3)  9 (10.5)  9 (10.5)  2 (2.3)  31 (36.0)  15 (17.4)  26 (30.2)  9 (10.5)  35 (40.7)  3 (3.8) (n = 80)  37 (43.0)  43 (50.0)  48 (55.8)  20 (23.3)  8 (7.1) (n = 85)  1 (1.2) (n = 85)  21 (25) (n = 84)  3 (3.5)  8 (9.3)  38 (44.2)  39 (45.3)  1 (1.2)  28 (32.6)  17 (19.8)  8 (9.3)  9 (10.5)  3 (3.5)  2 (2.3)  2 (2.3) | 67.1 ± 17.3  63 (72.4)  25.9 ± 6.6 (n = 82)  26 (29.9)  46 (52.9)  29 (33.3)  15 (17.9) (n = 84)  11 (12.6)  23 (26.4)  4 (4.6)  16 (18.4)  6 (6.9)  7 (8.0)  22 (25.3)  46 (54.1) (n = 85)  60 (69)  29 (33.3)  2 (2.3)  4 (4.6)  17 (19.5)  7 (8.0)  4 (4.6)  35 (40.2)  16 (18.4)  19 (21.8)  13 (14.9)  38 (43.7)  5 (6.0) (n = 84)  32 (36.8)  41 (47.1)  57 (65.5)  17 (19.5)  4 (4.8) (n = 84)  3 (3.5) (n = 85)  15 (18.8) (n = 80)  7 (8.3) (n = 84)  12 (13.8)  27 (31.0)  48 (55.2)  0 (0.0)  24 (27.6)  15 (17.2)  12 (13.8)  5 (5.7)  1 (1.1)  1 (1.1)  1 (1.1) | 67.5 ± 13.3  53 (64.6)  25.4 ± 5.2 (n = 80)  24 (29.3)  41 (50.0)  29 (35.4)  18 (22.8) (n = 79)  11 (13.4)  13 (15.9)  5 (6.1)  9 (11.0)  5 (6.1)  5 (6.1)  11 (13.4)  33 (31.6) (n = 79)  40 (48.8)  24 (29.3)  0 (0.0)  2 (2.4)  3 (3.7)  8 (9.8)  3 (3.7)  34 (41.5)  16 (19.5)  11 (13.4)  4 (4.9)  26 (31.7)  1 (1.3) (n = 79)  29 (35.4)  30 (36.6)  46 (56.1)  10 (12.2)  1 (1.3) (n = 80)  2 (2.5) (n = 80)  20 (24.4)  4 (4.9) (n = 81)  6 (7.3)  35 (42.7)  39 (47.6)  2 (2.3)  38 (46.3)  11 (13.4)  8 (9.8)  3 (3.7)  3 (3.7)  6 (7.3)  0 (0.0) | 0.58  0.27  0.26  0.80  0.99  0.98  0.02  0.79  < 0.01  0.58  0.07  0.55  0.49  0.20    < 0.01  < 0.01  0.69  0.76  0.11  < 0.01  0.93  0.88  0.81  0.54  0.04  0.16  0.43  0.23  0.75  0.25  0.02  0.049  0.35  0.65  0.16  0.54  0.14  0.02  0.34  0.10  0.18  0.72  0.15  0.17 | 0.17  0.06  0.10  0.87  0.95  0.77  0.02  0.49  0.80  0.70  0.15  0.25  0.87  0.12  0.36  0.71  0.78  0.33  0.69  0.99  0.87  0.75  0.98  0.37  0.41  0.75  0.42  0.75  0.43  0.65  0.03  0.75  0.18  0.36  0.14  0.59  0.59  0.85  0.26  0.053  0.77  0.71  0.09  0.73  0.84  0.27  0.03 |  |
|  |  |  |  |  |  |  |  |  |
|  |  |  |  |  |  |  |  |  |
|  |  |  |  |  |  |  |  |  |
|  |  |  |  |  |  |  |  |  |
|  |  |  |  |  |  |  |  |  |
|  |  |  |  |  |  |  |  |  |
|  |  |  |  |  |  |  |  |  |
|  |  |  |  |  |  |  |  |  |
|  |  |  |  |  |  |  |  |  |
|  |  |  |  |  |  |  |  |  |
|  |  |  |  |  |  |  |  |  |
|  |  |  |  |  |  |  |  |  |
|  |  |  |  |  |  |  |  |  |
|  |  |  |  |  |  |  |  |  |
|  |  |  |  |  |  |  |  |  |
|  |  |  |  |  |  |  |  |  |
|  |  |  |  |  |  |  |  |  |
|  |  |  |  |  |  |  |  |  |
|  |  |  |  |  |  |  |  |  |
|  |  |  |  |  |  |  |  |  |
|  |  |  |  |  |  |  |  |  |
|  |  |  |  |  |  |  |  |  |
|  |  |  |  |  |  |  |  |  |

ACE = angiotensin-converting enzyme, BB = betablockers, CCB = calcium channel blocker, COPD = chronic obstructive pulmonary disease, ICD = implantable cardioverter-defibrillator, NYHA = New York Heart Association, PCI = percutaneous coronary intervention, SD = standard deviation

| **Supplementary Table 3. Clinical, echocardiographic, and laboratory parameters according to** **initial CRP after exclusion of sepsis-triggered CS.** | | | | | | | | |
| --- | --- | --- | --- | --- | --- | --- | --- | --- |
|  | **Quartile 1  (< 8) (n = 86)** | **Quartile 2  (8 - 24) (n = 86)** | **Quartile 3  (24 - 56) (n = 87)** | **Quartile 4  (> 56) (n = 82)** | **p value** | **P_trend_** |  |  |
| Clinical presentation at admission  Heart rate, mean ± SD, bpm  SBP, mean ± SD, mmHg  DBP, mean ± SD, mmHg  MBP, mean ± SD, mmHg  Sinus rhythm, n (%)  Skin mottling, n (%)  Left heart failure, n (%)  Right heart failure, n (%)  Cardiac arrest, n (%)  Blood tests at admission, median (IQR)  Sodium, mmol/L  Potassium, mmol/L  Creatinin, μmol/L  Bilirubin, mg/L  Haemoglobin, g/dL  Arterial blood lactates, mmol/L  ASAT, UI/L  ALAT, UI/L  PT, %  Nt-proBNP, pg/mL  BNP, pg/mL  Baseline echocardiography  LVEF, mean ± SD, %  TAPSE, median (IQR), mm  PSVtdi, median (IQR), cm/s  Severe mitral regurgitation, n (%)  Severe aortic stenosis, n (%)  Severe aortic regurgitation, n (%) | 95.4 ± 33.4  103.9 ± 31.8  64.5 ± 20.0  76.5 ± 21.8  40 (46.5)  28 (37.3) (n = 75)  56 (65.1)  36 (41.9)  12 (14.0)  137.0 (134.0 – 140.0)  4.0 (3.7 – 5.0) (n = 78)  114.5 (92.3 – 149.0)  12.0 (7.2 – 20.4) (n = 70)  13.6 (12.0 – 15.0) (n = 85)  3.0 (2.0 – 5.7) (n = 77)  64.5 (38.8 – 165.3) (n = 70)  50.0 (26.3 – 115.0) (n = 74)  69.0 (49.3 – 88.3) (n = 84)  3,617.5 (736.0 – 7,418.5) (n = 32)  925.0 (239.5 – 1,422.3) (n = 38)  29.1 ± 14.6  15.0 (10.5 – 19.0) (n = 31)  8.0 (6.0 – 11.0) (n = 21)  9 (10.7) (n = 84)  5 (10.9)  2 (2.4) (n = 84) | 88.9 ± 25.9  100.0 ± 22.2  61.3 ± 14.0  73.7 ± 14.9 (n = 85)  51 (59.3)  18 (24.7) (n = 73)  64 (74.4)  56 (65.1)  3 (3.5)  135.5 (133.0 – 138.0)  4.2 (4.0 – 5.0) (n = 80)  140.5 (108.3 – 239.0)  20.0 (11.0 – 37.6) (n = 68)  13.0 (11.0 – 14.0)  2.7 (1.7 – 3.9) (n = 81)  48.0 (29.8 – 112.3) (n = 76)  37.0 (19.0 – 83.0) (n = 77)  54.0 (38.0 – 76.0) (n = 85)  12,652.0 (7,126.5 – 21,138.0) (n = 35)  1,564.0 (786.3 – 2,886.0) (n = 34)  26.0 ± 13.6  11.0 (9.0 – 15.0) (n = 39)  7.5 (6.0 – 10.5) (n = 30)  23 (27.7) (n = 83)  4 (4.7)  0 (0.0) | 94.6 ± 27.9  103.3 ± 23.5  64.6 ± 17.4  77.2 ± 18.1  36 (41.4)  27 (32.9) (n = 82)  69 (79.3)  54 (62.1)  3 (3.4)  135.0 (131.0 – 140.0)  4.3 (4.0 – 5.0) (n = 84)  142.0 (98.5 – 207.5)  21.0 (12.5 – 37.5) (n = 75)  13.0 (11.0 – 14.5)  3.0 (2.0 – 4.3) (n = 76)  116.0 (39.5 – 542.0) (n = 75)  61.0 (27.0 – 449.0) (n = 77)  49.0 (33.5 – 69.5) (n = 83)  11,517.0 (4,704.5 – 26,202.8) (n = 36)  1,688.0 (579.8 – 4,666.3) (n = 30)  24.0 ± 12.4 (n = 86)  13.0 (10.0 – 15.3) (n = 32)  7.0 (7.0 – 9.0) (n = 27)  18 (22.5) (n = 80)  5 (5.9) (n = 85)  3 (3.6) (n = 84) | 101.9 ± 32.7  98.3 ± 22.2  60.6 ± 16.4  73.1 ± 17.4  46 (56.1)  32 (47.1) (n = 68)  62 (75.6)  43 (52.4)  5 (6.1)  134.0 (130.3 – 138.0)  4.0 (4.0 – 5.0) (n = 74)  141.0 (101.5 – 210.3)  18.0 (10.1 – 30.5) (n = 70)  12.0 (10.0 – 13.5)  3.0 (2.0 – 7.8) (n = 74)  143.0 (78.3 – 492.8) (n = 66)  82.0 (37.0 – 258.5) (n = 67)  61.5 (43.8 – 78.0) (n = 74)  20,027.0 (6,059.0 – 35,000.0) (n = 32)  1,338.0 (816.8 – 3,190.5) (n = 34)  30.3 ± 14.4 (n = 78)  11.0 (9.0 – 13.0) (n = 27)  8.0 (6.0 – 10.0) (n = 25)  6 (10.3) (n = 78)  1 (1.3) (n = 80)  1 (1.3) (n = 78) | 0.08  0.52  0.49  0.48  0.07  0.04  0.18  < 0.01  0.02  < 0.01  0.20  < 0.01  < 0.01  < 0.01  0.06  < 0.01  < 0.01  < 0.01  < 0.01  < 0.01  < 0.01  0.07  0.87  < 0.01  0.44  0.34 | 0.09  0.64  0.51  0.74  0.68  0.16  0.09  0.22  0.049  < 0.01  0.16  0.03  < 0.01  < 0.01  0.76  < 0.01  0.01  0.03  < 0.01  < 0.01  0.98  0.03  0.66  0.49  0.23  0.95 |  |  |
|  |  |  |  |  |  |  |  |  |
|  |  |  |  |  |  |  |  |  |

ALAT = alanine aminotransferase, ASAT = aspartate aminotransferase, BB = betablockers, BNP = Brain natriuretic peptide, CRP = C-reactive protein, DBP = diastolic blood pressure, IQR = interquartile range, LVEF = left ventricular ejection fraction, MBP = mean blood pressure, Nt-proBNP = N-terminal-pro hormone BNP, PSVtdi = peak systolic velocity tissue Doppler imaging, PT = prothrombin time, SBP = systolic blood pressure, SD = standard deviation, TAPSE = tricuspid annular plane systolic excursion

| **Supplementary Table 4. In-hospital management** **according to initial CRP after exclusion of sepsis-triggered CS.** | | | | | | | |
| --- | --- | --- | --- | --- | --- | --- | --- |
|  | **Overall population  (n = 341)** | **Quartile 1  (< 8) (n = 86)** | **Quartile 2  (8 - 24) (n = 86)** | **Quartile 3  (24 - 56) (n = 87)** | **Quartile 4  (> 56) (n = 82)** | **p value** | **P_trend_** |
| Medications used, n (%)  Dobutamine  Norepinephrine  Epinephrine  Levosimendan  Loop diuretics  Thiazide diuretics  Aldosterone antagonist  Number of vasopressors/inotropes used, n (%)  0  1  2  3  4  Respiratory support, n (%)  Non-invasive  Invasive  Acute MCS, n (%)  Overall  IABP  Impella  ECLS  Renal replacement therapy, n (%)  Volume expander, n (%)  Isotonic saline  Balanced solutions  Glucose solution  Macromolecules  Any PCI, n (%)  Anti-infectious therapies, n (%)  Antibiotics  Antifungal agents | 267 (78.3)  170 (49.9)  39 (11.4)  30 (8.8)  233 (68.3)  13 (3.9) (n = 334)  48 (14.1)  38 (11.1)  138 (40.5)  130 (38.1)  32 (9.4)  3 (0.9)  62 (18.2)  101 (29.6)  50 (14.7)  19 (5.6)  10 (2.9)  27 (7.9)  43 (12.6)  123 (36.1)  77 (22.6)  17 (5.0)  53 (15.5)  13 (3.8)  111 (68.9) (n = 161)  82 (26.1) (n = 314)  5 (1.6) (n = 311) | 66 (76.7)  41 (47.7)  17 (19.8)  6 (7.0)  52 (60.5)  1 (1.2) (n = 84)  8 (9.3)  10 (11.6)  35 (40.7)  28 (32.6)  13 (15.1)  0 (0.0)  16 (18.6)  34 (39.5)  18 (20.9)  7 (8.1)  4 (4.7)  11 (12.8)  10 (11.6)  35 (40.7)  21 (24.4)  6 (7.0)  16 (18.6)  3 (3.5)  36 (75.0) (n = 48)  21 (25.6) (n = 82)  1 (1.2) (n = 83) | 69 (80.2)  38 (44.2)  7 (8.1)  11 (12.8)  64 (74.4)  4 (4.7)  16 (18.6)  9 (10.5)  37 (43.0)  34 (39.5)  4 (4.7)  2 (2.3)  13 (15.1)  19 (22.1)  11 (12.8)  2 (2.3)  2 (2.3)  7 (8.1)  11 (12.8)  21 (24.4)  14 (16.3)  2 (2.3)  7 (8.1)  2 (2.3)  24 (66.7) (n = 36)  9 (11.0) (n = 82)  1 (1.3) (n = 80) | 70 (80.5)  42 (48.3)  6 (6.9)  10 (11.5)  65 (74.7)  5 (6.0) (n = 83)  15 (17.2)  13 (14.9)  27 (31.0)  40 (46.0)  7 (8.0)  0 (0.0)  19 (21.8)  15 (17.2)  8 (9.2)  2 (2.3)  2 (2.3)  4 (4.6)  7 (8.0)  34 (39.1)  18 (20.7)  5 (5.7)  21 (24.1)  3 (3.4)  19 (55.9) (n = 34)  22 (28.6) (n = 77)  1 (1.3) (n = 75) | 62 (75.6)  49 (59.8)  9 (11.0)  3 (3.7)  52 (63.4)  3 (3.7) (n = 81)  9 (11.0)  6 (7.3)  39 (47.6)  28 (34.1)  8 (9.8)  1 (1.2)  14 (17.1)  33 (40.2)  13 (15.9)  8 (9.8)  2 (2.4)  5 (6.1)  15 (18.3)  33 (40.2)  24 (29.3)  4 (4.9)  9 (11.0)  5 (6.1)  32 (74.4) (n = 43)  30 (41.1) (n = 73)  2 (2.7) (n = 73) | 0.83  0.21  < 0.01  0.14  0.09  0.43  0.22  0.16  0.70  < 0.01  0.16  0.06  0.75  0.21  0.25  0.08  0.32  < 0.01  0.86 | 0.89  0.10  0.07  0.44  0.66  0.34  0.80  0.60  0.75  0.61  0.36  0.77  0.90  0.84  0.26  0.70  0.41  0.07  0.36  0.58  0.37  0.78  0.72  0.35  0.70  0.02  0.52 |

ECLS = extracorporeal life support, IABP = intra-aortic balloon pump, MCS = mechanical circulatory support, PCI = percutaneous coronary intervention

|  | |
| --- | --- |
| Quartile 1 Quartile 2 Quartile 3 Quartile 4 | |
| 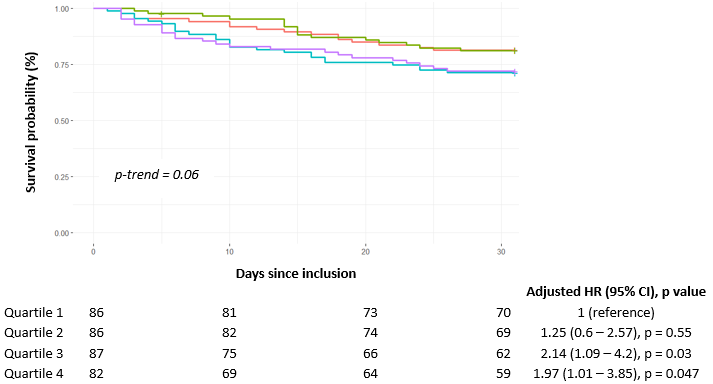 | 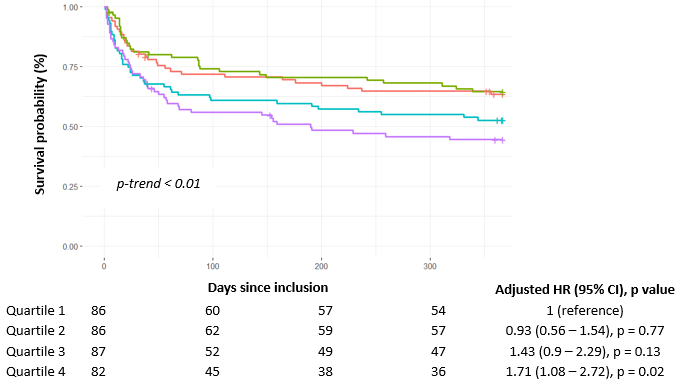 |

**SUPPLEMENTARY FIGURE 1: Short- and long-term mortality outcomes after CS according to baseline CRP after exclusion of sepsis-triggered CS.**

Panel A represents 1-month overall mortality. Panel B focus on 1-year mortality. The cumulative incidences of 1-year and 1-month mortality were estimated with the use of the Kaplan–Meier method; hazard ratios and 95% confidence intervals were estimated with the use of Cox regression models.

According to significant characteristics found as independent predictive factors in multivariable analyses, 1-year mortality was adjusted for age, previous PCI, SVT trigger, iatrogenic trigger. 1-month mortality was adjusted for age and current smoking.

CS = cardiogenic shock, HR = hazard ratio
