# Supplementary figures and images for: The admission level of CRP during cardiogenic shock is a strong independent risk marker of mortality
Source: Sci Rep. 2024 Jul 16;14:16338. doi: 10.1038/s41598-024-67556-y (PMC11252392; doi:10.1038/s41598-024-67556-y)

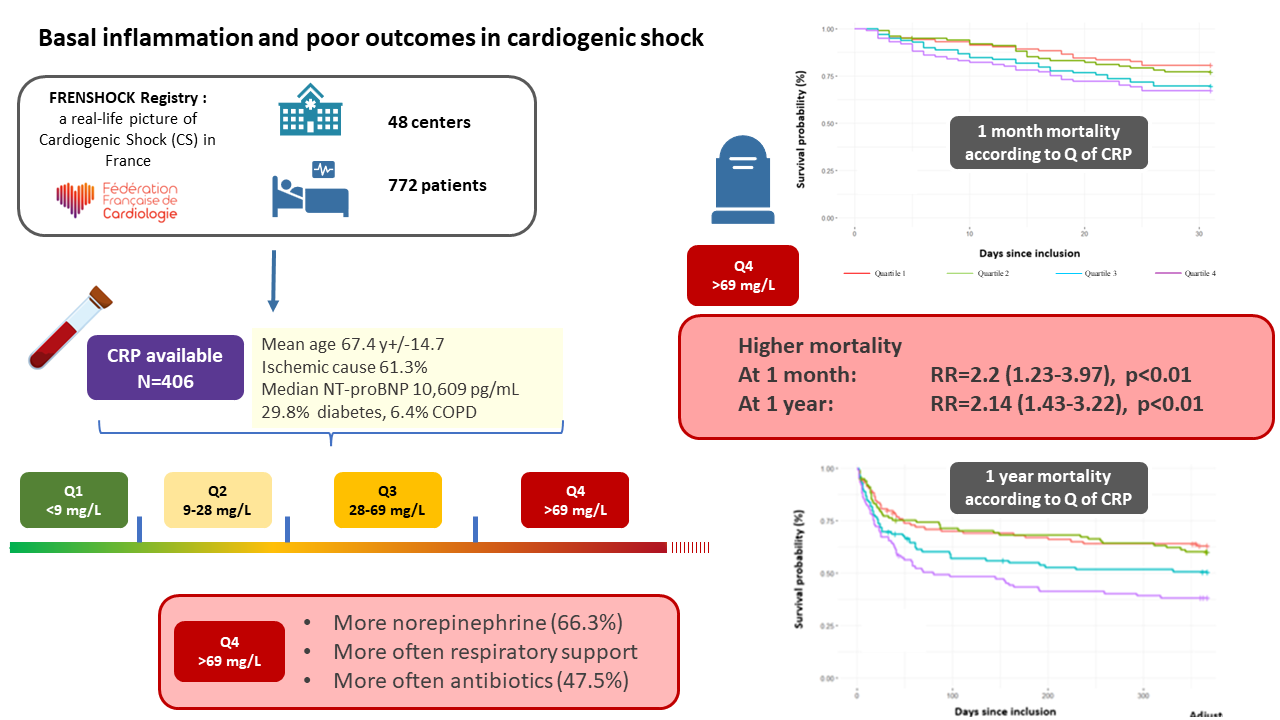

Supplement: Supplementary file 2 — Supplementary Information 2. [file 41598_2024_67556_MOESM2_ESM.png]
